# Gene Expression Deconvolution for Uncovering Molecular Signatures in Response to Therapy in Juvenile Idiopathic Arthritis

**DOI:** 10.1371/journal.pone.0156055

**Published:** 2016-05-31

**Authors:** Ang Cui, Gerald Quon, Alan M. Rosenberg, Rae S. M. Yeung, Quaid Morris, BBOP Study Consortium

**Affiliations:** 1 Division of Engineering Science, University of Toronto, Toronto, ON, Canada; 2 Department of Computer Science, University of Toronto, Toronto, ON, Canada; 3 Department of Pediatrics, Division of Rheumatology, University of Saskatchewan, Saskatoon, SK, Canada; 4 Divisions of Rheumatology and Cell Biology, The Hospital for Sick Children, Toronto, ON, Canada; 5 Departments of Paediatrics, Immunology and Medical Sciences, University of Toronto, Toronto, ON, Canada; 6 The Donnelly Centre, University of Toronto, Toronto, ON, Canada; 7 Department of Molecular Genetics, University of Toronto, Toronto, ON, Canada; University of Leuven, Rega Institute, BELGIUM

## Abstract

Gene expression-based signatures help identify pathways relevant to diseases and treatments, but are challenging to construct when there is a diversity of disease mechanisms and treatments in patients with complex diseases. To overcome this challenge, we present a new application of an *in silico* gene expression deconvolution method, ISOpure-S1, and apply it to identify a common gene expression signature corresponding to response to treatment in 33 juvenile idiopathic arthritis (JIA) patients. Using pre- and post-treatment gene expression profiles only, we found a gene expression signature that significantly correlated with a reduction in the number of joints with active arthritis, a measure of clinical outcome (Spearman rho = 0.44, p = 0.040, Bonferroni correction). This signature may be associated with a decrease in T-cells, monocytes, neutrophils and platelets. The products of most differentially expressed genes include known biomarkers for JIA such as major histocompatibility complexes and interleukins, as well as novel biomarkers including α-defensins. This method is readily applicable to expression datasets of other complex diseases to uncover shared mechanistic patterns in heterogeneous samples.

## Introduction

Juvenile idiopathic arthritis (JIA) is a family of heterogeneous autoimmune diseases characterized by chronic joint inflammation in children [1]. In JIA, prolonged joint inflammation leads to joint damage and subsequent functional disability [2–6]. However, the etiology of JIA remains unknown, and clinical parameters alone are inadequate to predict patient response to treatment. Consequently, there is a need to study the pathways affected by these diseases at the molecular level.

Molecular profiling has contributed to improved understanding of risk for progression and responses to treatment in JIA [7]. Transcriptional profiling for studies of immune diseases is often carried out on whole blood or peripheral blood mononuclear cells (PBMCs) as they likely include immune cells that reflect disease status, and are readily obtained by routine phlebotomy. Gene expression profiling has demonstrated the important role of specific cell types in JIA pathogenesis and identified potential mechanisms for clinical remission [8]. Expression profiling and genotyping have been used together to identify genetic or transcriptional variations associated with patient response to treatment [9]. In this study, gene expression profiles and clinical indicators of disease activity are measured pre- and post-treatment for multiple patients. Genes whose differences between pre- and post-treatment highly correlate with clinical response are candidate biomarkers of disease activity or targets for therapeutic intervention.

A major obstacle in identifying biomarkers for JIA treatment response arises from the difficulty in uncovering commonality across a population with diverse clinical profiles and patient-specific expression patterns. JIA encompasses diseases with a wide spectrum of clinical symptoms, progression and outcomes [10]. The International League of Associations for Rheumatology (ILAR) identified seven classes of JIA based on clinical symptoms at disease onset. Within each class, patients exhibit a wide variation with respect to disease course and treatment response. Furthermore, the specific treatment options and permutations of combined treatments are diverse, resulting in distinct expression signatures in each individual. This drives the need to develop a computational approach that combines expression profiles from a group of individuals with similar conditions to uncover the commonality in the course of treatment.

To identify common molecular signatures in a group of subjects, Quon et al. developed ISOpure-S1, a computational gene expression deconvolution method, to characterize a single, common cancer expression profile from heterogeneous tumor profiles [11, 12]. The method utilizes probabilistic algorithms to separate raw expression profiles into individual components, corresponding to the common cancer gene expression patterns across patients and healthy cells mixed into the tumor tissues. They found that ISOpure-S1’s estimation of cancer content in patient’s tissue was well correlated with pathologists’ estimates. Previous work has also demonstrated the robustness of similar deconvolution methods for inferring cellular composition of blood samples and identifying expression signatures in diseases [13–15].

In this pilot study, we present a new application of ISOpure-S1 to identify the common gene expression signature in response to treatment in JIA. We applied this method to whole blood samples drawn from 33 JIA patients pre-treatment and 6 months post-treatment to uncover the net effect of disease-modifying drugs. The model identified a common “treatment response” signature in all 33 patients despite their clinical heterogeneity. It then estimated a per-patient scalar parameter “% treatment response” that reflected the overall magnitude of the “treatment-response” signature observed in each post-treatment expression profile. We found the estimate for % treatment response significantly correlated with a clinical measure of treatment response. We also identified a list of differentially expressed genes that may aid in the understanding of JIA disease progression and treatment responses.

## Materials and Methods

### Ethics statement

This study is a part of the larger “Biologically-Based Outcome Predictors in JIA” (BBOP study) multi-center cohort study carried out in 11 Canadian academic health sciences centers. All of the institutional ethics review boards (listed in Acknowledgments) from each of the participating sites specifically approved this study. All participants provided their written consent to participate in this study using a paper-based consent form and process approved by each of the respective ethics review boards from each of the participating institutions. Informed written consent for a minor child was obtained from the child’s parent or legal guardian on behalf of the minor children enrolled in this study using consent forms and processes approved by the respective ethics review boards of each of the participating institutions. Informed written assent was obtained from minor children who were considered capable of providing assent. The assent form and processes for obtaining assent were approved by the respective ethics review boards of each of the participating institutions.

### Clinical samples

Children less than 16 years of age with new onset JIA were eligible for inclusion in this study. Detailed clinical and biological data were collected using uniform data collection instruments and standard operating procedures, including detailed clinical and gene expression data on each individual at baseline (time of diagnosis/study enrollment) and at 6 months after treatment. A proof of principle study on 33 paired patient samples at baseline and 6 months was performed. These 33 patients were diagnosed with different JIA subtypes as classified by ILAR criteria [1]. The study group comprised 18 polyarticular rheumatoid factor negative (RF-), seven polyarticular rheumatoid factor positive (RF+), five systemic, one oligoarticular, one psoriatic and one undifferentiated patients. The patients were recruited from the following sites: nine from BC Children’s Hospital, Vancouver, six from Montreal Children’s Hospital, six from Manitoba Health Sciences Center, four from The Hospital for Sick Children, Toronto, three from Janeway Hospital, St John’s, three from IWK Hospital, Halifax, one from the University of Saskatchewan, Saskatoon, and one from Stollery Children’s Hospital, Edmonton. There were 26 female patients in the dataset (79%). Table 1 summarizes the clinical characteristics of the patient cohort. Four patients had been treated with disease-modifying anti-rheumatic drugs (DMARDs) prior to the pre-treatment blood draw and therefore their pre-treatment profiles were excluded from the study. 15 of the 29 DMARD-naïve patients received non-steroidal anti-inflammatory drugs (NSAIDs) prior to the pre-treatment blood draw. Between the baseline and 6-month blood draws, patients, in addition to receiving an NSAID, were prescribed one or more of the following drugs: Cyclosporin, Hydroxychloroquine, Methotrexate, Sulfasalazine (DMARDs); Anakinra, Etanercept (Biologic therapies); and/or Prednisone.

**Table 1:** Measures of disease activity in patient cohort.

	0 months	6 months
**Physician’s global assessment (PGA), median (IQR)**	4.9 (2.1–6.4)	1.2 (0.1–2.2)
**Erythrocyte sedimentation rate (ESR), median (IQR)**	20 (8–54)	9 (3–16)
**Number of active joints, median (IQR)**	10 (6–18)	2 (0–10)
**Number of LRM (limited range of motion) joints, median (IQR)**	5 (3–14)	1 (0–3)

Internationally accepted measures of disease activity including those in the ACR pediatric core set at baseline and 6 months.

### Microarray data collection

Blood samples were collected, transported and processed in accordance with strict standard operating procedures. Briefly, peripheral blood was collected in Tempus Blood RNA Tubes (Applied Biosystems), which contain a proprietary reagent to lyse blood cells upon collection and permit stable storage and transportation of total RNA and transported to a central processing site within 5 days [16]. RNA was purified at a central biorepository using proprietary buffer according to the manufacturer’s instructions using the Tempus Spin RNA Isolation Reagent Kit (Applied Biosystems). RNA samples were assayed for quality (A260:A280 and A260:A230 ratios >1.8; RIN >7.0; Agilent BioAnalyzer) and concentration prior to labeling and processing. Whole genome gene expression profiles were determined using Illumina Human HT-12 Expression BeadChip Arrays. Each array targets more than 47,000 probes designed to cover genes from NCBI RefSeq Release 38 as well as legacy UniGene genes.

### Computational approach

ISOpure was previously developed to improve the analysis accuracy of tumor expression profiles [12]. It consists of two steps; here only the first step (ISOpure-S1) was used to capture a common change in expression profiles across all JIA patients, with which the biomarkers associated with response to treatment were identified.

**Fig 1:**
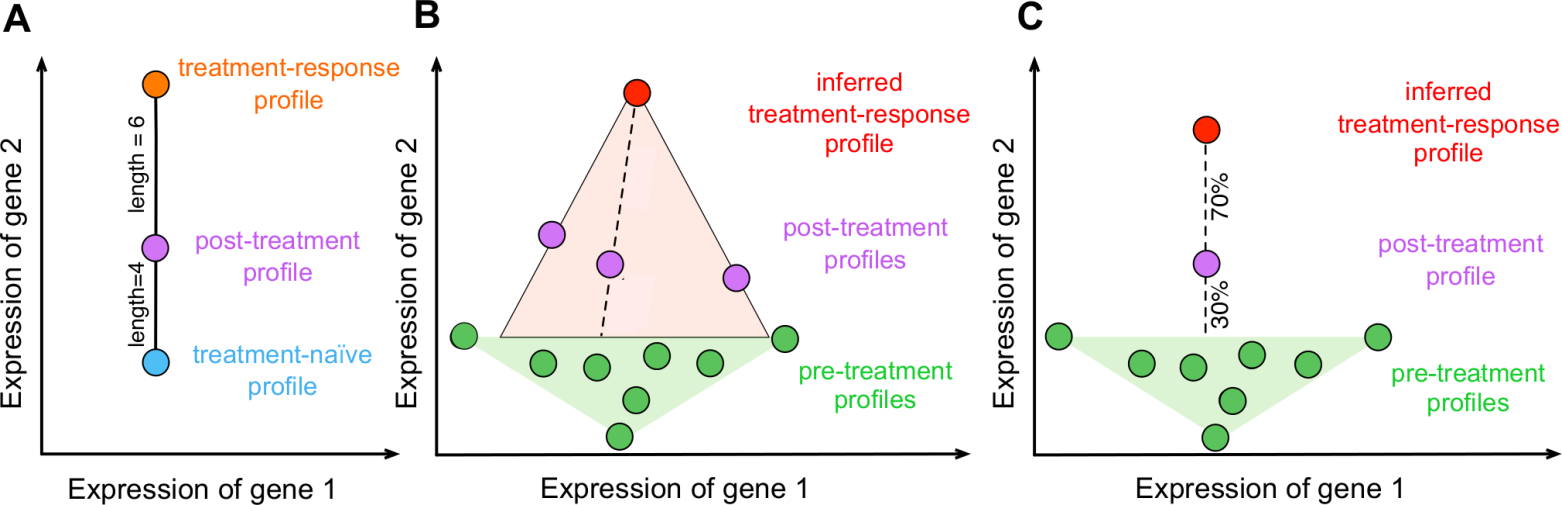
Graphical representation of the ISOpure-S1 algorithm. The ISOpure-S1 algorithm is based on a probabilistic topic model. We modeled post-treatment gene expression profiles as mixtures of hidden profiles corresponding to treatment-response and treatment-naive portions, and removed the gene expression signals of the treatment-naive portion using pre-treatment expression profiles. (A) We modeled the post-treatment expression profile as a weighted average of the gene expression profiles of treatment-response and treatment-naive profiles. (B) We inferred a single common treatment-response profile after drug treatment across all patients that corresponds to the portion of the post-treatment profiles that cannot be attributed to any pre-treatment profiles. (C) We used this inferred treatment-response profile to estimate the % treatment-response content in each patient. Steps (B) and (C) were done iteratively during the maximum likelihood estimation procedure.

A geometric intuition for our model is shown in Fig 1. ISOpure-S1 deconvolves, or separates, each post-treatment expression profile into a treatment-naïve component and a treatment-response component, where the treatment-naïve component is estimated from a linear combination of all patients’ pre-treatment profiles. For each post-treatment expression profile, ISOpure-S1 identifies a “residual” gene expression pattern that cannot be explained by any combination of the pre-treatment profiles. This residual profile thus comprises patterns due to treatment response as well as measurement noise. ISOpure-S1 estimates a single common treatment-response profile that best explains all of the residuals observed for each of the post-treatment profiles. The gene expression patterns that cannot be attributed to pre-treatment profiles or the treatment-response profile are considered individual variations in response to treatment and/or noise and not modeled. The model also computes a “% treatment response” scalar per patient, which represents the predicted size of the treatment response in the post-treatment profile. Because ISOpure-S1 deconvolves post-treatment profiles using the pre-treatment profiles of all patients for which these data are available, this treatment-response profile automatically excludes patient-specific signatures. Finally, the genes targeted by the treatment can be predicted by a delta profile, which is obtained by comparing the treatment-response profile to the pre-treatment profiles.

ISOpure-S1 takes as input *M (= 29)* pre-treatment and *N (= 33)* post-treatment gene expression profiles (ISOpure-S1 does not require the numbers of pre- and post-treatment profiles to be equal). Four pre-treatment profiles were removed because the patients were taking DMARDs prior to their first blood draws. The model outputs four parameters of interest: 1) a single treatment-response gene expression profile common to all patients, 2) a % treatment-response estimate for each post-treatment expression profile, 3) a single delta profile that captures the difference between the treatment-response profile and the pre-treatment profiles, and 4) a list of genes predicted to be differentially expressed under treatment in the delta profile. The MATLAB commands for running ISOpure-S1 are included as S1 File.

### Model description

In the following model description, variables are in italics, constants are in upper case, vectors are in bold, and index references are in italic lowercased letters. The notation [***u v***] represents a matrix constructed by horizontally appending column vectors and/or matrices ***u*** and ***v***. The notation ***Mv*** represents matrix-vector product of ***M*** and ***v***. The notation $\hat{p}$ represents the estimation of variable ***p*.**

The input to ISOpure-S1 is a set of expression profiles ***b***_***m***_ from *M* pre-treatment blood samples, and ***p***_***n***_ from *N* post-treatment blood samples.

Let the matrices ***B*** and ***P*** be defined as

$$B=[b_{1}\ldots \;b_{M}]$$

$$P=[p_{1}\ldots \;p_{N}]$$

where each pre-treatment profile ***b***_***m***_ and post-treatment profile ***p***_***n***_ is composed of non-negative expression measurements from *G* genes. ISOpure-S1 estimates a single, non-negative treatment-response profile denoted as ***r***_._ ***b***_***m*,**_ ***p***_***n***_ and ***r*** are column vectors with *G* elements. Our model requires the vector ***p***_***n***_ to be a “count” vector that contains non-negative integers; if this vector is initially real-valued, it can be re-scaled and discretized to achieve the desired precision in representation of the gene expression levels. Each pre-treatment profile ***b***_***m***_ is scaled such that all its *G* entries sum to 1 and, as such, it can be interpreted as discrete probability distribution over transcripts. The treatment-response profile, ***r***, inferred by the model also sums to 1 and permits a similar interpretation.

ISOpure-S1 models each post-treatment profile as a non-negative, convex combination (mixture) of pre-treatment profiles in ***B*** and the treatment-response profile ***r***, and uses *θ*_*n*,*d*_, where *d* ε {1, …, *M*+1}, to indicate the convex weight of these profiles. The ***θ***_***n***_ vector is restricted such that the (*M*+1) entries in each ***θ***_***n***_ sum to 1, so that ***θ***_***n***_ corresponds to a discrete probability distribution over profiles.

$${\hat{p}}_{n}=\left[B\;r\right]\theta_{n}$$

$$P(p_{n}|\;B,r,\theta_{n})∼Multinomial\;(p_{n}|{\hat{p}}_{n})$$

The hidden variables ***θ***_***n***_ are initialized such that all entries are equal to 1/(*M*+1). ***θ***_***n***_ are inferred using *Maximum A Posteriori* (MAP) estimation from a Dirichlet prior ***v***, which is initialized to be a set of random numbers greater than 1 for the first 1 to *M* entries, and greater than 5 for the (*M*+1)th entry. The (*M*+1)th entry is the proportion of the expression profile associated with the treatment-response profile. We assign a larger prior for this proportion because there are *M* profiles capturing the treatment-naive portion and only one profile capturing the treatment-response portion.

$${P(\theta }_{n}|\;v)∼Dirichlet\;(\theta_{n}|\;v)$$

The treatment-response profile, ***r***_,_ is also inferred using MAP from a Dirichlet prior, which is constructed by a convex combination of pre-treatment profiles ***B***. The convex weights are denoted by ***ω*** and the strength between the treatment-response profile and its prior is denoted as *κ*, which is initialized to 10,000.

P(r|κ,ω,B)∼Dirichlet(r|κωB)

The complete log likelihood is defined as:

lnL=lnP(p,θ,r|v,κ,ω,B)

$$=\text{ln}\;P\left(r|\;\kappa ,\omega ,B\right)+\sum_{n=1}^{N}[\text{ln}\;P(\theta_{n}|v)+\;\text{ln}\;P(p_{n}|\;B,r,\theta_{n})]$$

***θ***_***d***_, ***r***, ***v***, ***ω*** and *κ* are estimated by maximizing the complete log likelihood function. 20 iterations of the optimization procedure were performed and yielded a relative change in log likelihood of less than 10^−7^ between the final two iterations. Each iteration procedure used the Polak-Ribière conjugate gradient descent method to estimate variables of the same type simultaneously (where we assigned the same letter to variables of the same type). To find a good local (and possibly global) maximum, 10 random initializations were performed and the one that achieves the highest complete likelihood was used.

To identify the differentially expressed genes, a delta profile, **δ**, was computed by comparing the treatment-response profile and a convex combination of pre-treatment profiles.

$$\delta ={log}_{2}\left(r\right)-\;{log}_{2}\left(\omega B\right)$$

The genes with the highest fold changes (log2 differences) in the delta profile were identified as the differentially expressed genes after treatment.

### Sensitivity test

The model sensitivity analysis was performed by dividing the total set of profiled genes into four subsets and applying ISOpure-S1 on each subset separately. The robustness was measured based on the standard error of the model parameters estimated on the subsets of the data.

### Cell type analysis

To uncover the cell types associated with treatment response, a Spearman correlation test was performed between each delta profile and cell type profile published by Novershtern et al [17]. The probes were first centered by their mean across all samples before measuring Spearman correlation.

## Results

### Impact of treatment on gene expression is correlated with disease activity

**Fig 2:**
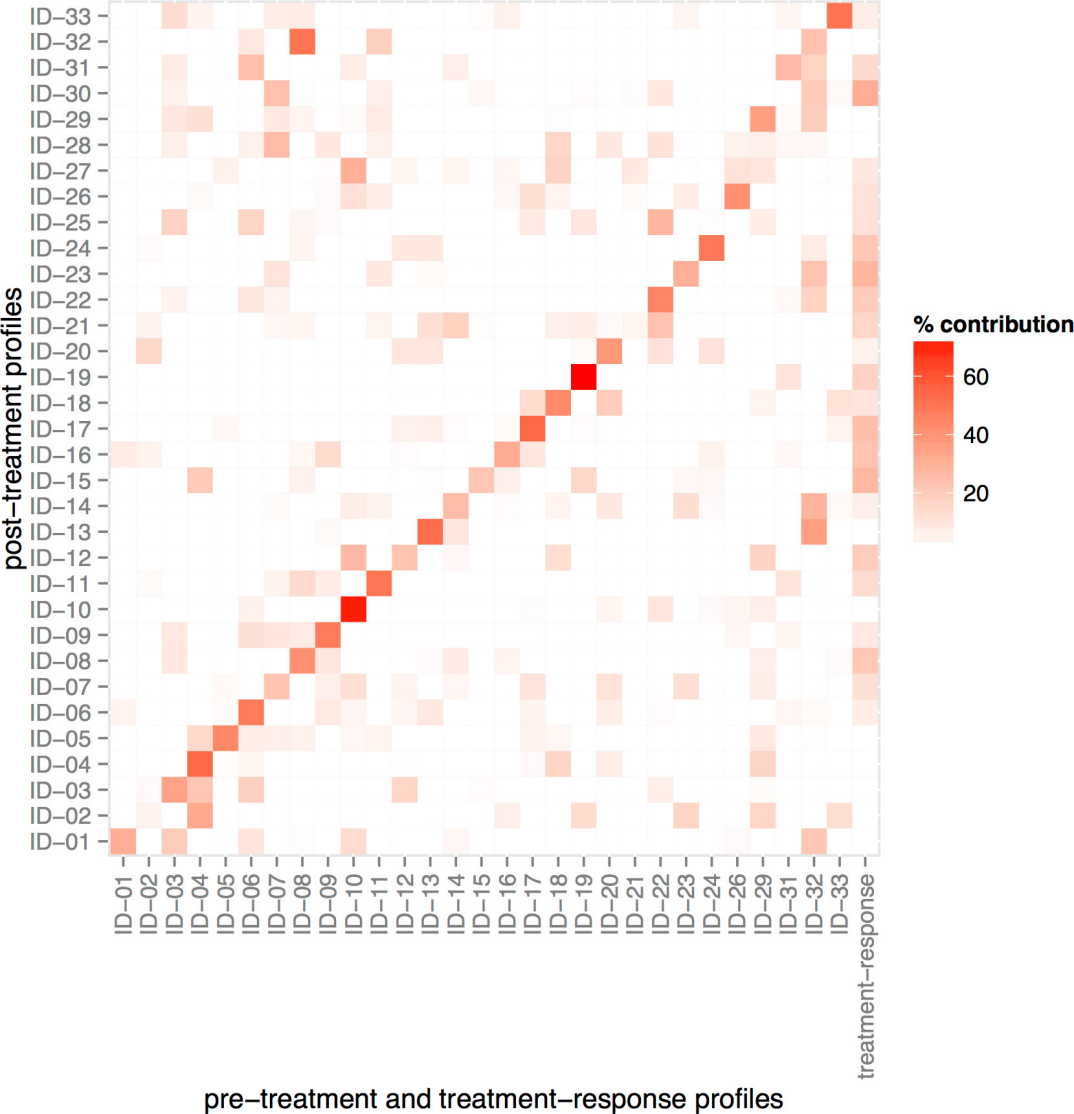
ISOpure-S1-inferred compositions of post-treatment profiles in terms of pre-treatment profiles and “treatment-response” profile. Each post-treatment profile was attributed to a combination of the pre-treatment profiles (column 1–29) and the “treatment-response” profile (column 30). The shading represents the percentage contribution from each of the pre-treatment profiles and the “treatment-response” profile.

We applied ISOpure-S1 to the 33 post-treatment profiles using the 29 pre-treatment profiles as a reference panel. Using *Maximum A Posteriori* (MAP) estimation, ISOpure-S1 simultaneously fits 1) an inferred treatment-response profile, and 2) 30 non-negative weights for each of the post-treatment profiles. These weights describe a convex combination of the 29 pre-treatment profiles and the inferred treatment-response profile (in other words, the weights are all between 0 and 1 and they sum to one) (Fig 2). Each weight corresponds to the proportion of the post-treatment profile explained by each of the 30 components (29 profiles in the reference panel and the one inferred treatment-response profile; the ‘% treatment response’ scalar is the weight of the treatment-response profile). For 23 of the 29 patients, the model automatically assigned their pre-treatment profile the highest weight in the deconvolution of their post-treatment profiles, despite the fact that the algorithm did not use sample pairing information. This result suggests that the strong, patient-specific gene expression patterns in raw expression profiles are removed in the inferred treatment-response profile.

**Fig 3:**
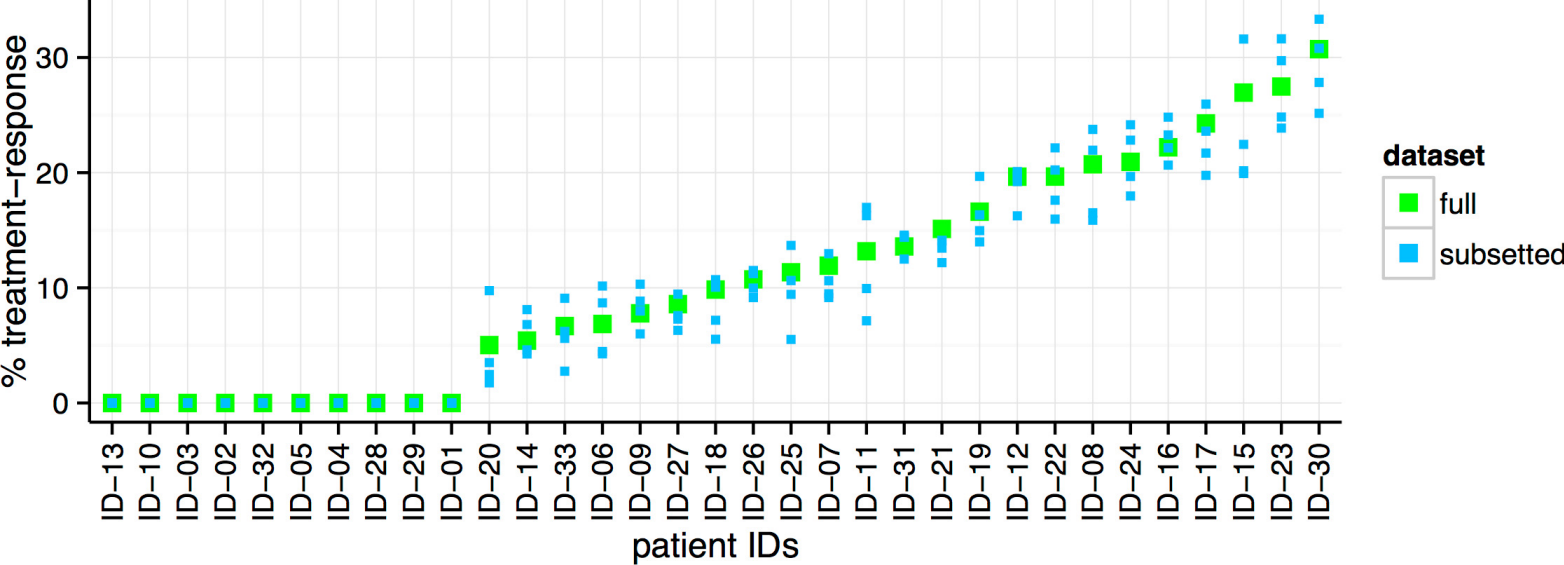
The per-patient % treatment-response estimates made by ISOpure-S1, sorted by increasing order (green dots). A sensitivity analysis was performed to verify the robustness of our results (blue dots), which was done by dividing the total set of profiled genes into four subsets and applying ISOpure-S1 on each subset separately. The % treatment-response estimates for each subset of the profile (blue dots) are similar to the original results (green dots) (average standard error = 0.019; maximum standard error = 0.057).

There is wide variation in the % treatment-response estimates for the 33 patients (Fig 3). To confirm that the estimates are robust and not driven by small variations in the dataset, we performed a sensitivity test. We divided the total set of profiled genes into four subsets, and applied ISOpure-S1 on each subset separately. The % treatment-response estimates for each subset of the profile (blue dots) are similar to the original results (green dots), suggesting that the model is indeed robust (average standard error = 0.019; maximum standard error = 0.057).

**Fig 4:**
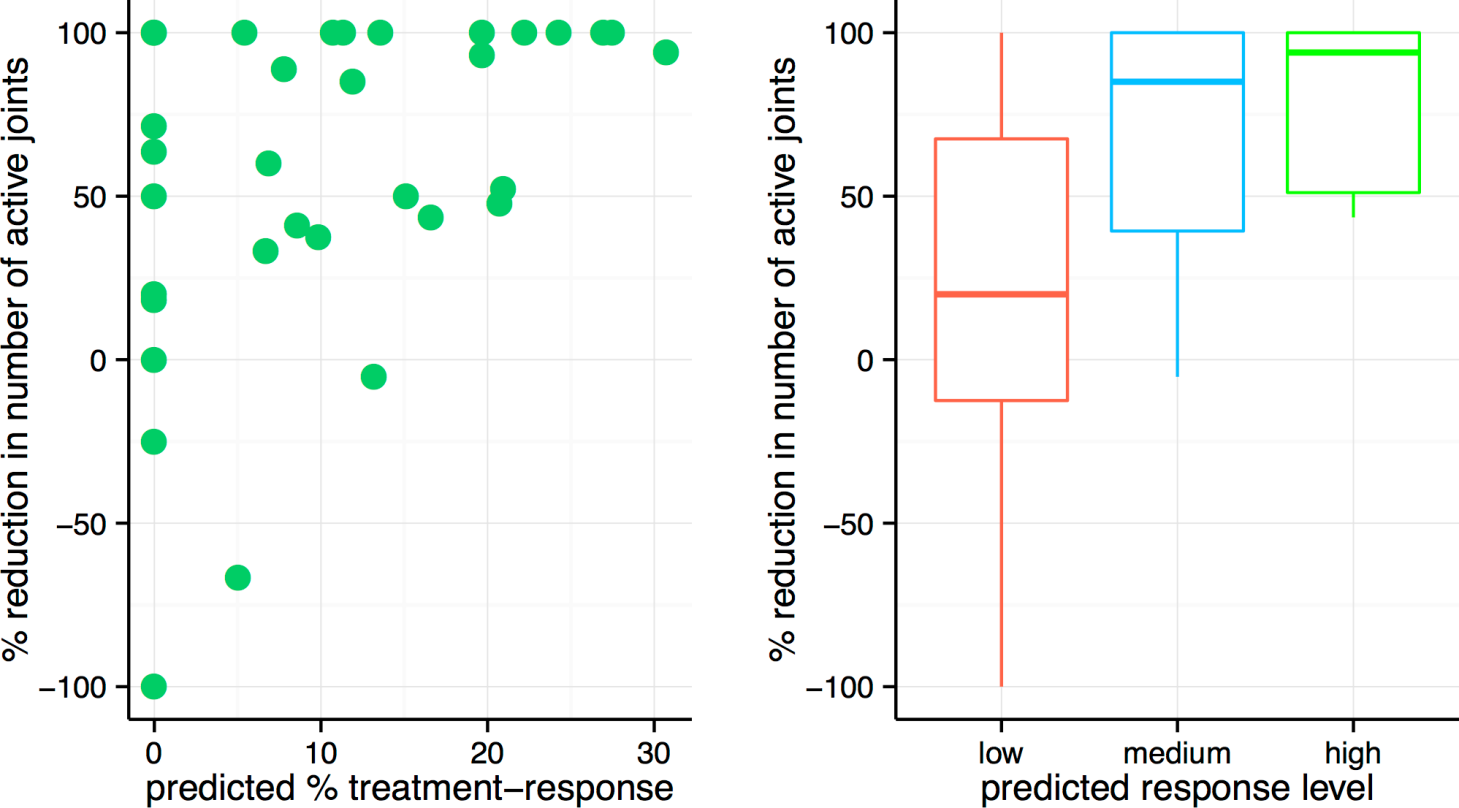
Correlation between expression-based % treatment-response estimate inferred by ISOpure-S1 and % reduction in number of active joints found in clinical record. Left: Correlation between % treatment-response estimate and % reduction in number of active joints (Pearson rho = 0.44, p = 0.010; Spearman rho = 0.46, p = 0.008). Right: The patients were divided evenly into three groups based on their % treatment-response estimates. The median and interquantile range of % reduction in number of active joints are indicated by box plots.

Next, we compared our % treatment-response estimates with clinical indicators of disease activity. We measured four individual components of the validated ACR (American College of Rheumatology) pediatric core set measure of disease activity for JIA, including physician’s global assessment (PGA), erythrocyte sedimentation rate (ESR), % reduction in number of joints with active arthritis (active joints), and % reduction in joints with limited range of motion (LRM joints) [18]. These four clinical indicators are uncorrelated with each other except for the % reduction in active joint count and the % reduction in LRM joint count (S1 Table). We found that the % treatment-response estimate significantly correlated with a % reduction in number of active joints (Spearman rho = 0.44, p = 0.040; Pearson rho = 0.46, p = 0.032, Bonferroni correction) (Table 2, Fig 4). Individuals with high % treatment response consistently showed a decrease in the number of active joints, whereas the change in % of active joints was much more variable for individuals with low % treatment response (Fig 4, right panel). We recognized that systemic JIA patients might have more distinct disease mechanisms compared with the rest of the cohort. Therefore, we repeated this analysis without the five systemic JIA patients. The trend remained the same for the non-systemic JIA cohort (Spearman rho = 0.49, p = 0.032; Pearson rho = 0.48, p = 0.036, Bonferroni correction) (S1 Fig).

**Table 2:** Correlation between ISOpure-S1-predicted % treatment-response and % decrease in clinical indicators of disease activity.

Clinical indicator of disease activity	Correlation with % treatment-response estimates (Spearman)	Bonferroni-corrected p-value	Correlation with % treatment-response estimates (Pearson)	Bonferroni-corrected p-value
**PGA**	-0.037	1.000	-0.124	1.000
**ESR**	0.292	0.396	0.158	1.000
**# Active Joints**	0.443	0.040 *	0.457	0.032 *
**# LRM Joints**	0.114	1.000	0.173	1.000

* p<0.05

### Genes influenced by treatment are enriched for immune functions

**Fig 5:**
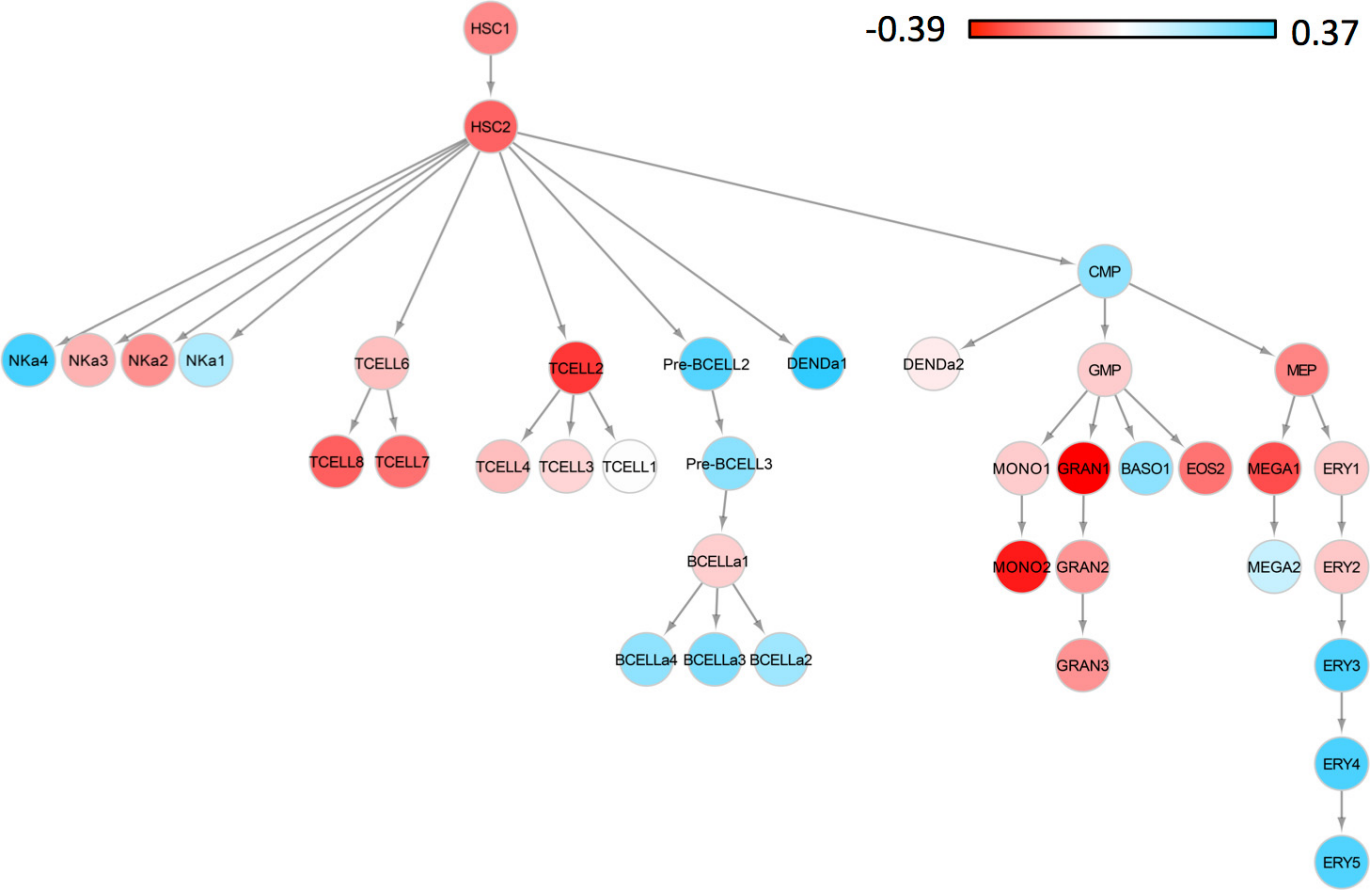
A decrease in the proportion of T-cells, neutrophils, monocytes and platelets in blood may be associated with a response to drug treatment. We correlated the delta profile with the profiles of 38 known blood cell types. The red color indicates a decrease in their concentrations and the blue color indicates an increase in their concentrations in blood in response to drug treatment. The color bar indicates the Spearman correlation coefficients.

To identify the cell types affected by the treatment of JIA, we compared our delta profile against the profiles of 38 known blood cell types [17]. First, we confirmed the absence of batch effects by calculating the correlation between the raw differences in each pair of patient profiles and the reference blood cell type profiles (S2 Fig). These two types of profiles did not cluster by study, indicating that the patient profiles from this study are comparable with reference blood cell type profiles. We then correlated the 38 reference blood cell types with the delta profile, which represents the changes in gene expression patterns associated with response to treatment. In this analysis, we assumed that the gene expression profile of each cell type remained relatively constant. However, it is worth noting that the drug treatments most likely impacted both cell type composition, as well as expression patterns in individual cell types. The delta profile is negatively correlated with the profiles of neutrophils, monocytes, T-cells and megakaryocytes (progenitor of platelets) suggesting that the decrease of these cell types in the blood may be associated with response to treatment (Fig 5). To verify this result, we examined the change in the cell counts measured in phlebotomy. Indeed, there was a 4.05% median decrease in white blood cell count (11.33% decrease in neutrophils and 5.00% decrease in lymphocytes) and 2.25% decrease in platelet count. In particular, there was a strong positive correlation between changes in platelet count and in the number of active joints (Spearman rho = 0.52, p = 0.009).

We identified the differentially expressed genes associated with treatment response by extracting the top up- and down-regulated genes in the delta profile (Table 3 and Page 1 in S2 Table). We performed functional enrichment analysis (DAVID) [19, 20] on the top 100 up- and down-regulated genes (S2 File), and found that these genes are enriched for immune functions (GO terms include immune response, defense response, and inflammatory response). The down-regulated genes contain markers of immune activation and are involved in host defense to infectious organisms, while the up-regulated genes contain those related to leukocyte recruitment and soluble mediators of the innate immunity. Many down-regulated genes also show a large decrease in the average raw expression values (Page 1 in S2 Table).

**Table 3:** Top 20 up- and down-regulated genes identified by ISOpure-S1.

Up-regulated genes		Down-regulated genes	
Gene symbol	Fold change	Gene symbol	Fold change
LOC731682	2.65	DEFA3	-6.08
PDZK1IP1	2.34	DEFA1B*	-6.06
FCER1A	2.26	LOC649923	-5.96
IL8	2.05	DEFA1	-5.90
IL8RB	1.78	DEFA2*	-5.76
D4S234E	1.72	MGC29506	-5.74
NFXL1	1.63	IGJ	-5.70
MFF	1.59	ZDHHC19	-5.58
PTPRC	1.51	IFI44L	-5.37
FOS	1.51	LOC652775	-5.22
HOPX	1.50	IGLL1	-5.20
PI3	1.50	IFI27	-5.14
JMJD1C	1.49	ISG15	-5.10
ITM2B	1.49	TNFRSF17	-5.03
FFAR2	1.45	LOC652493	-4.95
MGC72104	1.43	C19ORF59	-4.90
LOC643319	1.41	ELA2	-4.79
MYL4	1.40	TXNDC5	-4.73
FAM126B	1.37	LOC652694	-4.67
DUSP1	1.37	HES4	-4.65

A list of the differentially expressed genes in the delta profile (impacted by treatment) and their fold changes (log2 differences).

* Reclassified as DEFA1

We summarized the functions of some up- and down-regulated genes that ISOpure identifies (Tables 4 and 5). A number of interleukin (IL) and IL receptor genes, including IL8, IL8RB (CXCR2), IL6R, and IL2RB were significantly over-expressed (among the top 100 up-regulated genes). Among the most down-regulated genes in our study, we also found well-known biomarkers for autoimmune diseases: the major histocompatibility complex class II (MHC II), DR beta (HLA-DRB). We also found some newer targets with very strong signals: α-defensins and type 1 interferon-inducible proteins. The α-defensin genes DEFA1, 1B, 2, 3 and 4 were all among the top 21 down-regulated genes. The most up- and down-regulated genes in the analysis of non-systemic JIA patients were similar: all the genes in Tables 4 and 5 were among the top 600 down- and up-regulated genes in the non-systemic JIA analysis.

**Table 4:** Selected highly up-regulated genes identified by ISOpure-S1 and their families.

Up-regulated genes after treatment	Differential expression fold changes [Rank]	Protein families
PDZK1IP1	2.34 [2]	PDZK1 Interacting protein
IL8	2.05 [4]	Interleukin
IL8RB (CXCR2)	1.78 [5]	
IL2RB	1.12 [66]	
IL6R	1.09 [77]	
PTPRC	1.51 [9]	Protein Tyrosine Phosphatase

Rank of fold change (log2 difference) in sorted delta profile is indicated in bracket.

**Table 5:** Selected highly down-regulated genes identified by ISOpure-S1 and their families.

Down-regulated genes after treatment	Differential expression fold changes [Rank]	Protein families
DEFA3	-6.08 [1]	α-defensin
DEFA1B*	-6.06 [2]	
DEFA1	-5.90 [4]	
DEFA2*	-5.76 [5]	
DEFA4	-4.60 [21]	
IFI44L	-5.37 [9]	Type 1 interferon inducible gene product
IFI27	-5.14 [12]	
ISG15	-5.10 [13]	
HLA-DRB1	-3.06 [91]	Major histocompatibility complex, class II, DR beta
HLA-DRB5	-2.16 [218]	
HLA-DRB3	-2.02 [265]	
IL4R	-3.30 [67]	Interleukin
IL17RA	-2.07 [244]	
IL1R2	-2.00 [280]	
IL18RAP	-1.89 [326]	

Rank of fold change (log2 difference) in sorted delta profile is indicated in bracket.

* Reclassified as DEFA1

In contrast, the top 100 most significantly differentially expressed genes obtained by t-test (Page 2 in S2 Table) on the original expression profiles are not enriched for immune functions according to DAVID. These genes do not overlap with the top 100 up- and down-regulated genes obtained by ISOpure-S1. The gene product of most differentially expressed genes from the t-test include opioid receptor, ADP-ribosylation factor, calcium/calmodulin-dependent protein kinase, zinc-finger protein, and tumor necrosis factor (TNF) receptor. It is worth noting that TRAF6, a molecule involved in TNF receptor family signaling and known to be associated with multiple autoimmune diseases including rheumatoid arthritis [21], was one of the most differentially expressed genes, indicating that a decrease in TRAF6 expression level may be associated with treatment effects in JIA.

## Discussion

We explored the utility of ISOpure-S1 for uncovering a treatment-response profile in blood samples of JIA patients with different symptoms and treatments. We did this by deconvolving heterogeneous blood expression profiles using only whole blood samples. We found that many patient-specific expression signatures were successfully removed after deconvolution. The resulting % treatment-response estimates significantly correlated with a reduction in the number of joints with active arthritis, a clinical indicator of treatment response, despite the small cohort size, heterogeneity in medication use, and the absence of clinical information as model input. The common defining feature of the group of diseases described as JIA is joint inflammation (i.e. arthritis); therefore in spite of the heterogeneity of other clinical features among the different types of JIA the correlation with the % decrease in the number of joints with active arthritis is expected. The differentially expressed genes in the associated delta profile were enriched for immune functions, and we found that α-defensins and type 1 interferon-inducible proteins were likely impacted by treatment in JIA. The enrichment of immune function genes among differentially expressed genes is consistent with the central role of immune activation in inflammatory arthritis and the known mechanism of action of anti-inflammatory medications used in these children.

Our results show that a global decrease in T-cell, neutrophil, monocyte and platelet concentrations may be associated with response to treatment. Monocytes and neutrophils are important components of the innate immune system and are involved in signaling the adaptive immune system to respond to antigens. A decrease in the activation level of T-cells may indicate reduced inflammatory response, or adaptive immune response towards self-antigens, and may therefore be important to clinical remission of autoimmune diseases. A decrease in platelet count in response to treatment is also consistent with prior findings [22]. In the future, with better resolution of cell types (e.g. subtypes of T-cells, including Th1, Th17) our approach could again be used to identify which specific cell types are most involved in the treatment response.

The molecular signature we found in response to treatment suggests ongoing pro-inflammatory activity. While many pro-inflammatory markers were down-regulated after treatment, other pro-inflammatory markers such as IL8 were up-regulated. This is not surprising because Jarvis et al. showed persistent *in vitro* pro-inflammatory activity even in patients who were in remission [8]. They postulated that remission might be a state of homeostasis when the effects of the anti-inflammatory network predominated over the effect of the pro-inflammatory network. As evident in our study, the absolute values of fold changes (log2 differences) of most down-regulated genes were much higher than those of the most up-regulated genes. This is consistent with the notion that the expression of genes that are up-regulated in active JIA is decreased by drug treatment.

We identified a number of differentially expressed genes in the blood samples between pre- and post-treatment individuals. These differentially expressed genes include HLA-DRB and several interleukin and interleukin receptor genes that are known biomarkers for autoimmune diseases. HLA-DRB gene products are involved in antigen presentation and are up-regulated with activation of the adaptive immune response. The reduction in expression of MHC II is also in keeping with down-regulation of the T-cell levels that we observed. The identified interleukins are soluble mediators of inflammation associated with leukocyte recruitment and the innate immune response [23–26]. IL6 is a well-known soluble mediator of inflammation involved in the pathogenesis of arthritis and is currently a target for treatment in both children and adults with arthritis. IL8 is a chemotactic factor that selectively recruits lymphocytes and neutrophils and is an expression signature in JIA [27, 28]. The presence of these well-known biomarkers improves our confidence that the genes identified by our model are involved in JIA disease processes. Interestingly, the most differentially expressed genes identified by our method and by the t-test did not overlap. This shows that our method can be used to complement traditional methods when identifying differential expression signatures.

We also identified two families of genes not previously described as associated with treatment response in JIA: α-defensins and type 1 interferons. α-defensins are homologous peptides expressed by cells participating in the innate immune response including neutrophils, macrophages and intestinal Paneth cells [29]. In addition to their role in innate immunity, recent studies showed that α-defensins may also play a role in the pathogenesis of autoimmune diseases [30]. DEFA1-3 (α-defensins 1–3) increase secretion of pro-inflammatory molecules and enhance the presentation of costimulatory molecules on T-cells [31, 32]. Previous studies indicated that defensins were dysregulated in rheumatoid arthritis compared to control samples [33]; our analysis showed that a decrease in the expression of defensin genes in peripheral blood may be linked to the impact of treatment in JIA. Out of the 6 α-defensins found in humans, the ones we identified (DEFA1-4) are the only ones that are expressed in human neutrophils. Type 1 interferons gene products have been associated with autoimmune diseases such as systemic lupus erythematosus [34]. Our results showed that type 1 interferon gene products may be impacted by treatment in JIA. Recent studies showed that IFN-β was detrimental to Th17 mediated autoimmunity [35], and a review by Espinosa et al. indicated that the inflamed joints in JIA patients expressed high levels of IL17-producing T-cells [36]. Together, these may support the association between levels of type 1 interferon-inducible gene products and arthritis disease activities observed in some patients in our study. Other genes identified by our model, listed in Table 3 and Page 1 in S2 Table, may also be targets for future research.

There are some limitations in this study. Firstly, although the % decrease in active joint count is a readily quantifiable measure of a good treatment response in an individual patient, this measure of effect size does not account for expected variation in the counts (e.g. statistical significance). For example, decreases in active joint counts from 30 to 15 and from 2 to 1 both represent a 50% decrease, even though we intuitively have higher confidence in the decrease from 30 to 15 joints. Secondly, although we have explained a common treatment response profile for a majority of the patients, a few who we predicted to have poor treatment response had good clinical recovery (Fig 4). This pilot study motivates future studies to incorporate multiple treatment-response profiles into the model when larger patient cohort is available. Finally, this study motivates the use of experimental approaches to study and validate the functional impact of the differentially expressed genes we reported in arthritis treatment response.

## Conclusion

We applied a novel statistical deconvolution method (ISOpure-S1) to capture a shared molecular signature correlated with a measure of patient outcome in 33 JIA patients having varied characteristics, clinical symptoms and treatments. The results indicated that a decrease in T-cells, monocytes and neutrophils might correlate with a clinical improvement. Furthermore, we identified a list of novel genes that may be impacted by treatments in JIA, including α-defensins. ISOpure-S1 can readily be applied to heterogeneous gene expression profiles in case control, and pre- and post-treatment studies to investigate the pathogenesis and/or disease progression of other complex diseases. The method is especially valuable to study rare diseases that may share common mechanisms by aggregating a small number of patients despite their heterogeneous characteristics.

## Supporting Information

S1 Fig Correlation between expression-based % treatment-response estimate inferred by ISOpure-S1 and % reduction in number of active joints in non-systemic JIA patients. The analysis in Fig 4 is repeated here with the five systemic JIA patients removed, leaving a cohort of patients with more homogeneous clinical presentations.
(PDF)

S2 Fig The batch effect between patient profiles and reference blood cell type profiles is minimal. We computed the raw differences between each pair of patient profiles, and then calculated the correlation between the raw differences of patient profiles and the reference blood cell type profiles. The Spearman correlation is indicated by color.
(PDF)

S1 File MATLAB script to run ISOpure-S1. MATLAB script including a description of input and output data.
(PDF)

S2 File DAVID functional enrichment analysis. Functional annotation on the top 100 down- and up-regulated genes after treatment.
(PDF)

S1 Table Spearman correlations between clinical indicators of disease activity. (PDF)

S2 Table Differentially expressed genes identified by ISOpure-S1 and the standard t-test. ISOpure-S1 results (page 1) and the standard t-test results (page 2).
(XLSX)

## Abbreviations

ACR: American College of Rheumatology
BBOP: Biologically-Based Outcome Predictors in JIA Consortium
DMARD: disease-modifying anti-rheumatic drug
ESR: erythrocyte sedimentation rate
ILAR: International League of Associations for Rheumatology
JIA: juvenile idiopathic arthritis
LRM: limited range of motion
MHC: major histocompatibility complex
NSAID: non-steroidal anti-inflammatory drugs
PBMC: peripheral blood mononuclear cell
PGA: physician’s global assessment
RF: rheumatoid factor
Th: T helper subset (e.g. Th1, Th17)
