# Supplementary figures and images for: Gene Expression Deconvolution for Uncovering Molecular Signatures in Response to Therapy in Juvenile Idiopathic Arthritis
Source: PLoS One. 2016 May 31;11(5):e0156055. doi: 10.1371/journal.pone.0156055 (PMC4887077; doi:10.1371/journal.pone.0156055)

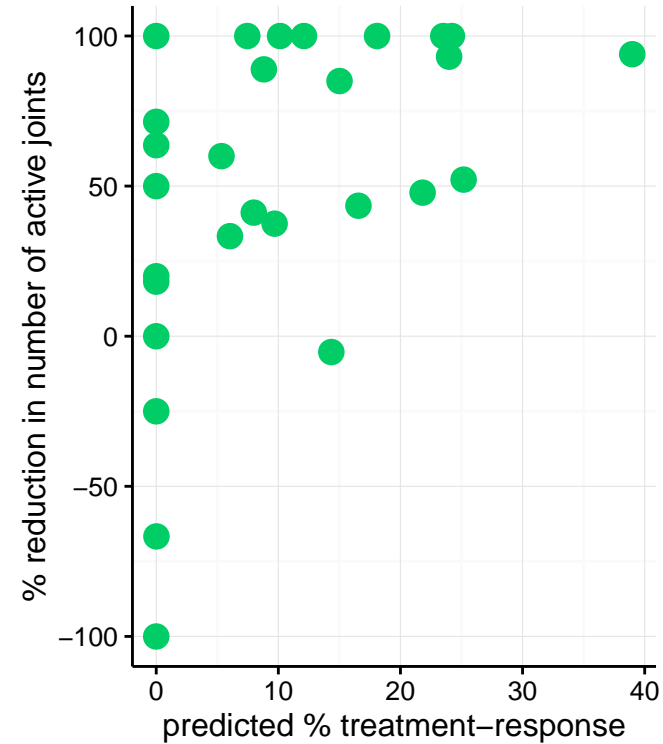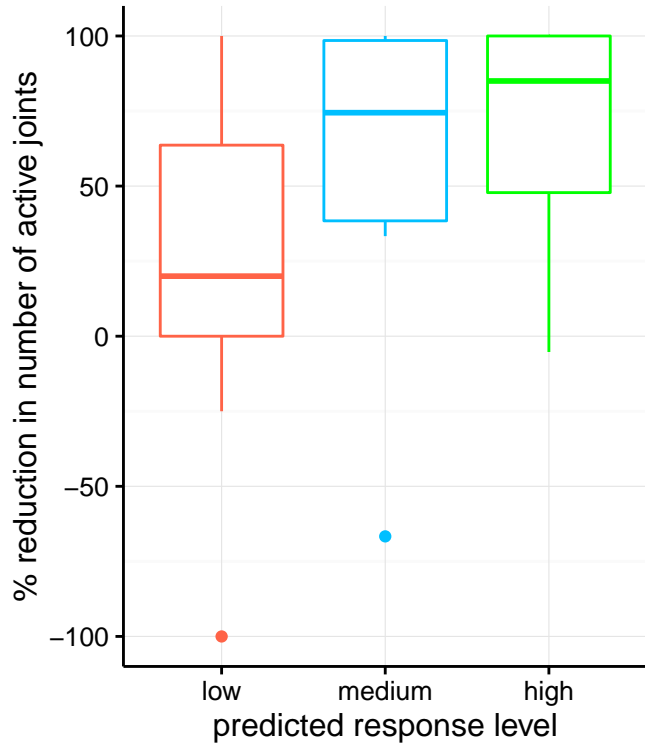

Supplement: S1 Fig — The analysis in Fig 4 is repeated here with the five systemic JIA patients removed, leaving a cohort of patients with more homogeneous clinical presentations. (PDF) [file pone.0156055.s001.pdf]

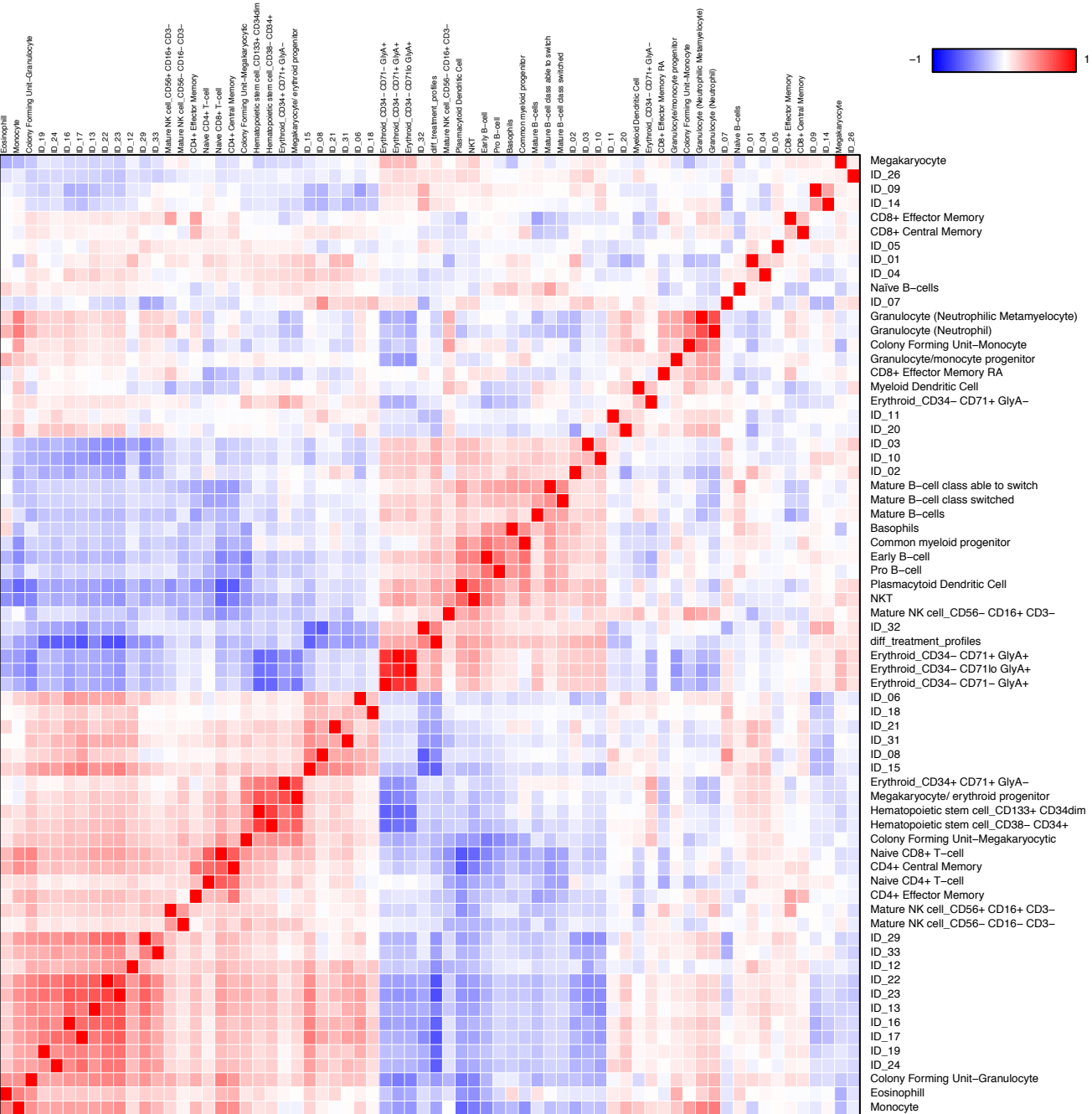

Supplement: S2 Fig — We computed the raw differences between each pair of patient profiles, and then calculated the correlation between the raw differences of patient profiles and the reference blood cell type profiles. The Spearman correlation is indicated by color. (PDF) [file pone.0156055.s002.pdf]
