# Supplementary material for: Gene Expression Deconvolution for Uncovering Molecular Signatures in Response to Therapy in Juvenile Idiopathic Arthritis
Source: PLoS One. 2016 May 31;11(5):e0156055. doi: 10.1371/journal.pone.0156055 (PMC4887077; doi:10.1371/journal.pone.0156055)
Supplement: S1 File — MATLAB script including a description of input and output data. (PDF) [file pone.0156055.s003.pdf]

MATLAB script to run ISOpure-S1

% ISOpure-S1 code can be obtained from https://github.com/gquon/ISOpure

% INPUT:
% PP: a GxN matrix representing gene expression profiles of post-treatment samples, where G is the number of genes and N is the number of subjects.
% BB: a GxM matrix representing gene expression profiles of pre-treatment samples, where M is the number of subjects in pre-treatment samples.

% OUTPUT:
% loglikelihood: log likelihood of the final model
% S1model: a structure with the following important fields:
% S1model.theta: an Nx(M+1) matrix, giving the fractional composition of each post-treatment profile. Each row represents a post-treatment sample that was part of the input. The first M columns correspond to the fractional compositions with respect to the pre-treatment profiles. The last column represents the fractional composition of the treatment-response profile.
% S1model.alphapurities: an Nx1 vector representing the proportion of treatment response (same as the last column of the theta variable; pulled out for user convenience).
% S1model.mm: a Gx1 vector representing the treatment-response profile, in the form of parameters of a multinomial or discrete distribution (sum of elements is 1).
% S1model.omega: an Mx1 vector describing the convex combination weights learned by ISOpureS1 over the BB matrix that form the prior over the treatment-response profile.
% S1model.PPtranspose: transpose of the normalized BB matrix such that the weights of G genes sum to 1 for each sample

% The following code assumes the variables are already loaded: BB, PP (see above), genes (vector of IDs of genes corresponding to the rows of PP and BB)

% Add ISOpure-S1 to user library

```
addpath(genpath('<path to ISOpureS1>'))
```

% Run ISOpure-S1

```
[S1model loglikelihood] = learnmodel(PP, BB)
```

% Output 1: a single treatment-response profile common to all patients

```
S1model.mm
```

% Output 2: a % treatment-response estimate for each post-treatment expression profile

S1model.alphapurities

% Output 3: a single delta profile that captures the difference between the treatment-response profile and the pre-treatment profiles

deltaprofile = log2(S1model.mm)-log2(S1model.PPtranspose' * S1model.omega)

% Output 4: a list of genes predicted to be differentially expressed under treatment in the delta profile

[result sorted_overexpr_ix] = sort(deltaprofile, 'descend');

sorted_genes = genes(sorted_overexpr_ix); % where “genes” is a vector containing gene symbols

CUTOFF = 100;

up_reg_genes = sorted_genes(1:CUTOFF)

down_reg_genes = sorted_genes(size(sorted_genes,1)-CUTOFF+1:size(sorted_genes,1))
