# Supplementary material for: Gene Expression Deconvolution for Uncovering Molecular Signatures in Response to Therapy in Juvenile Idiopathic Arthritis
Source: PLoS One. 2016 May 31;11(5):e0156055. doi: 10.1371/journal.pone.0156055 (PMC4887077; doi:10.1371/journal.pone.0156055)
Supplement: S2 File — Functional annotation on the top 100 down- and up-regulated genes after treatment. (PDF) [file pone.0156055.s004.pdf]

DAVID functional enrichment analysis with the top 100 down- and up-regulated genes

Down-regulated genes:

Sublist	Category	Term	RT	Genes	Count	%	P-Value	Benjamini
	GOTERM_BP_FAT	defense response to bacterium	RT		8	1.2	1.6E-6	1.3E-3
	GOTERM_BP_FAT	response to bacterium	RT		8	1.2	5.5E-5	2.2E-2
	GOTERM_BP_FAT	immune response	RT		13	1.9	1.7E-4	4.6E-2
	KEGG_PATHWAY	Hematopoietic cell lineage	RT		5	0.7	7.4E-4	4.8E-2
	GOTERM_BP_FAT	defense response to fungus	RT		3	0.4	1.9E-3	3.3E-1
	GOTERM_BP_FAT	killing of cells of another organism	RT		3	0.4	2.2E-3	3.1E-1
	GOTERM_BP_FAT	defense response	RT		10	1.5	3.9E-3	4.1E-1
	GOTERM_BP_FAT	cell killing	RT		3	0.4	6.6E-3	5.4E-1
	GOTERM_BP_FAT	response to fungus	RT		3	0.4	6.6E-3	5.4E-1
	GOTERM_BP_FAT	positive regulation of locomotion	RT		4	0.6	1.4E-2	7.6E-1
	GOTERM_BP_FAT	regulation of locomotion	RT		5	0.7	1.7E-2	7.8E-1
	GOTERM_BP_FAT	response to virus	RT		4	0.6	1.8E-2	7.8E-1
	GOTERM_MF_FAT	antigen binding	RT		3	0.4	2.1E-2	9.9E-1
	KEGG_PATHWAY	Intestinal immune network for IgA production	RT		3	0.4	2.4E-2	5.5E-1
	GOTERM_MF_FAT	copper ion binding	RT		3	0.4	3.1E-2	9.6E-1
	GOTERM_BP_FAT	membrane organization	RT		6	0.9	4.5E-2	9.7E-1
	GOTERM_BP_FAT	defense response to Gram-negative bacterium	RT		2	0.3	5.0E-2	9.7E-1
	GOTERM_MF_FAT	carbohydrate binding	RT		5	0.7	5.3E-2	9.8E-1
	GOTERM_BP_FAT	regulation of cell migration	RT		4	0.6	5.5E-2	9.7E-1
	GOTERM_MF_FAT	oxidoreductase activity, acting on the CH-NH2 group of donors	RT		2	0.3	7.0E-2	9.8E-1
	GOTERM_BP_FAT	positive regulation of cell migration	RT		3	0.4	7.6E-2	9.9E-1
	GOTERM_BP_FAT	regulation of cell motion	RT		4	0.6	7.6E-2	9.9E-1

	GOTERM_BP_FAT	amino acid transport	RT		3	0.4	7.7E-2	9.8E-1
	GOTERM_MF_FAT	neurotransmitter:sodium symporter activity	RT		2	0.3	7.7E-2	9.7E-1
	GOTERM_BP_FAT	positive regulation of cell motion	RT		3	0.4	8.9E-2	9.9E-1
	GOTERM_MF_FAT	neurotransmitter transporter activity	RT		2	0.3	9.2E-2	9.7E-1

Up-regulated genes:

Sublist	Category	Term	RT	Genes	Count	%	P-Value	Benjamini
	GOTERM_BP_FAT	anti-apoptosis	RT		8	1.0	7.6E-5	9.1E-2
	GOTERM_BP_FAT	inflammatory response	RT		9	1.1	2.1E-4	1.3E-1
	GOTERM_BP_FAT	positive regulation of cell communication	RT		9	1.1	2.3E-4	9.3E-2
	GOTERM_BP_FAT	response to wounding	RT		11	1.3	2.9E-4	8.9E-2
	GOTERM_BP_FAT	regulation of apoptosis	RT		13	1.6	6.0E-4	1.4E-1
	GOTERM_BP_FAT	regulation of programmed cell death	RT		13	1.6	6.5E-4	1.3E-1
	GOTERM_BP_FAT	regulation of cell death	RT		13	1.6	6.7E-4	1.1E-1
	GOTERM_BP_FAT	defense response	RT		11	1.3	9.4E-4	1.4E-1
	GOTERM_BP_FAT	positive regulation of response to stimulus	RT		7	0.9	1.2E-3	1.5E-1
	GOTERM_BP_FAT	regulation of response to external stimulus	RT		6	0.7	1.2E-3	1.4E-1
	GOTERM_BP_FAT	positive regulation of immune system process	RT		7	0.9	1.2E-3	1.3E-1
	GOTERM_BP_FAT	response to oxidative stress	RT		6	0.7	1.4E-3	1.4E-1
	GOTERM_BP_FAT	positive regulation of MAP kinase activity	RT		5	0.6	1.7E-3	1.5E-1
	GOTERM_BP_FAT	negative regulation of apoptosis	RT		8	1.0	2.0E-3	1.6E-1
	GOTERM_BP_FAT	response to protein stimulus	RT		5	0.6	2.0E-3	1.6E-1
	GOTERM_BP_FAT	positive regulation of cellular biosynthetic process	RT		11	1.3	2.1E-3	1.5E-1
	GOTERM_BP_FAT	negative regulation of programmed cell death	RT		8	1.0	2.1E-3	1.4E-1

Sublist	Category	Term	RT	Genes	Count	%	P-Value	Benjamini
	GOTERM_BP_FAT	negative regulation of cell death	RT		8	1.0	2.1E-3	1.4E-1
	GOTERM_BP_FAT	positive regulation of biosynthetic process	RT		11	1.3	2.3E-3	1.4E-1
	GOTERM_BP_FAT	response to abiotic stimulus	RT		8	1.0	2.4E-3	1.4E-1
	GOTERM_BP_FAT	protein kinase cascade	RT		8	1.0	2.5E-3	1.4E-1
	GOTERM_BP_FAT	response to organic substance	RT		11	1.3	3.1E-3	1.6E-1
	GOTERM_BP_FAT	response to radiation	RT		6	0.7	3.3E-3	1.7E-1
	GOTERM_BP_FAT	positive regulation of macromolecule metabolic process	RT		12	1.5	3.4E-3	1.6E-1
	GOTERM_BP_FAT	positive regulation of signal transduction	RT		7	0.9	3.6E-3	1.7E-1
	GOTERM_BP_FAT	response to inorganic substance	RT		6	0.7	3.7E-3	1.6E-1
	GOTERM_BP_FAT	positive regulation of response to external stimulus	RT		4	0.5	4.1E-3	1.7E-1
	GOTERM_BP_FAT	positive regulation of catalytic activity	RT		9	1.1	4.4E-3	1.8E-1
	GOTERM_BP_FAT	cytokine-mediated signaling pathway	RT		4	0.5	5.2E-3	2.0E-1
	GOTERM_BP_FAT	regulation of MAP kinase activity	RT		5	0.6	5.5E-3	2.1E-1
	GOTERM_BP_FAT	response to gamma radiation	RT		3	0.4	5.9E-3	2.1E-1
	GOTERM_BP_FAT	response to reactive oxygen species	RT		4	0.5	6.3E-3	2.2E-1
	GOTERM_BP_FAT	response to cytokine stimulus	RT		4	0.5	7.3E-3	2.4E-1
	GOTERM_BP_FAT	positive regulation of multicellular organismal process	RT		6	0.7	7.6E-3	2.5E-1
	GOTERM_BP_FAT	activation of MAPK activity	RT		4	0.5	8.1E-3	2.5E-1
	GOTERM_BP_FAT	regulation of protein kinase cascade	RT		6	0.7	8.2E-3	2.5E-1
	GOTERM_BP_FAT	positive regulation of molecular function	RT		9	1.1	8.9E-3	2.6E-1
	GOTERM_BP_FAT	response to molecule of bacterial origin	RT		4	0.5	9.2E-3	2.6E-1
	GOTERM_BP_FAT	positive regulation of chemotaxis	RT		3	0.4	9.3E-3	2.6E-1
	GOTERM_BP_FAT	regulation of cellular protein metabolic process	RT		8	1.0	9.5E-3	2.6E-1
	GOTERM_BP_FAT	regulation of leukocyte activation	RT		5	0.6	9.7E-3	2.6E-1

Sublist	Category	Term	RT	Genes	Count	%	P-Value	Benjamini
	GOTERM_BP_FAT	positive regulation of protein kinase cascade	RT		5	0.6	9.9E-3	2.6E-1
	GOTERM_BP_FAT	positive regulation of anti-apoptosis	RT		3	0.4	9.9E-3	2.5E-1
	GOTERM_BP_FAT	regulation of chemotaxis	RT		3	0.4	1.1E-2	2.6E-1
	GOTERM_BP_FAT	negative regulation of macromolecule metabolic process	RT		10	1.2	1.1E-2	2.6E-1
	GOTERM_BP_FAT	cellular ion homeostasis	RT		7	0.9	1.1E-2	2.6E-1
	GOTERM_BP_FAT	mitochondrial membrane organization	RT		3	0.4	1.1E-2	2.6E-1
	GOTERM_BP_FAT	regulation of cell activation	RT		5	0.6	1.2E-2	2.6E-1
	GOTERM_BP_FAT	cellular chemical homeostasis	RT		7	0.9	1.2E-2	2.7E-1
	GOTERM_BP_FAT	locomotory behavior	RT		6	0.7	1.2E-2	2.6E-1
	GOTERM_BP_FAT	positive regulation of behavior	RT		3	0.4	1.3E-2	2.7E-1
	GOTERM_BP_FAT	negative regulation of cellular protein metabolic process	RT		5	0.6	1.3E-2	2.7E-1
	GOTERM_BP_FAT	positive regulation of developmental process	RT		6	0.7	1.3E-2	2.6E-1
	GOTERM_BP_FAT	regulation of cytokine production	RT		5	0.6	1.3E-2	2.6E-1
	GOTERM_BP_FAT	negative regulation of protein metabolic process	RT		5	0.6	1.4E-2	2.8E-1
	GOTERM_BP_FAT	cell activation	RT		6	0.7	1.5E-2	2.8E-1
	GOTERM_BP_FAT	positive regulation of nitrogen compound metabolic process	RT		9	1.1	1.5E-2	2.8E-1
	GOTERM_BP_FAT	regulation of anti-apoptosis	RT		3	0.4	1.6E-2	2.9E-1
	GOTERM_BP_FAT	regulation of locomotion	RT		5	0.6	1.6E-2	2.9E-1
	GOTERM_BP_FAT	positive regulation of leukocyte activation	RT		4	0.5	1.6E-2	2.9E-1
	GOTERM_BP_FAT	regulation of protein modification process	RT		6	0.7	1.6E-2	2.9E-1
	GOTERM_BP_FAT	positive regulation of macromolecule biosynthetic process	RT		9	1.1	1.6E-2	2.9E-1
	GOTERM_BP_FAT	regulation of cell proliferation	RT		10	1.2	1.7E-2	2.8E-1
	GOTERM_BP_FAT	ion homeostasis	RT		7	0.9	1.7E-2	2.8E-1
	GOTERM_BP_FAT	regulation of MAPKKK cascade	RT		4	0.5	1.7E-2	2.9E-1

Sublist	Category	Term	RT	Genes	Count	%	P-Value	Benjamini
	GOTERM_BP_FAT	positive regulation of cell activation	RT		4	0.5	1.8E-2	3.0E-1
	GOTERM_MF_FAT	structural molecule activity	RT		9	1.1	1.9E-2	9.9E-1
	GOTERM_BP_FAT	response to cAMP	RT		3	0.4	1.9E-2	3.0E-1
	KEGG_PATHWAY	Jak-STAT signaling pathway	RT		5	0.6	1.9E-2	6.4E-1
	GOTERM_BP_FAT	cellular response to oxidative stress	RT		3	0.4	2.0E-2	3.1E-1
	GOTERM_BP_FAT	myeloid leukocyte activation	RT		3	0.4	2.2E-2	3.4E-1
	GOTERM_BP_FAT	regulation of behavior	RT		3	0.4	2.2E-2	3.4E-1
	GOTERM_BP_FAT	cellular response to stress	RT		8	1.0	2.3E-2	3.4E-1
