# Supplementary material for: Gene Expression Deconvolution for Uncovering Molecular Signatures in Response to Therapy in Juvenile Idiopathic Arthritis
Source: PLoS One. 2016 May 31;11(5):e0156055. doi: 10.1371/journal.pone.0156055 (PMC4887077; doi:10.1371/journal.pone.0156055)
Supplement: S1 Table — (PDF) [file pone.0156055.s005.pdf]

S1 Table. Spearman correlations between clinical indicators of disease activity.

	PGA	ESR	# active joints	# LRM joints
**PGA**	--	--	--	--
**ESR**	- 0.002 (p=0.993)	--	--	--
**# active joints**	- 0.157 (p=0.386)	- 0.115 (p=0.523)	--	--
**# LRM joints**	- 0.328 (p=0.076)	- 0.269 (p=0.150)	0.696 (p=1.9e-5*)	--

* p < 0.01
